# The integration of MRI in radiation therapy: collaboration of radiographers and radiation therapists

**DOI:** 10.1002/jmrs.225

**Published:** 2017-02-16

**Authors:** Robba Rai, Shivani Kumar, Vikneswary Batumalai, Doaa Elwadia, Lucy Ohanessian, Ewa Juresic, Lynette Cassapi, Shalini K. Vinod, Lois Holloway, Paul J. Keall, Gary P. Liney

**Affiliations:** ^1^ Liverpool and Macarthur Cancer Therapy CentresLiverpoolNew South WalesAustralia; ^2^ Ingham Institute for Applied Medical ResearchLiverpoolNew South WalesAustralia; ^3^ South Western Sydney Clinical SchoolUniversity of New South WalesLiverpoolNew South WalesAustralia; ^4^ Western Sydney UniversitySydneyNew South WalesAustralia; ^5^ Centre for Medical Radiation PhysicsUniversity of WollongongLiverpoolNew South WalesAustralia; ^6^ School of PhysicsUniversity of SydneySydneyNew South WalesAustralia; ^7^ School of MedicineUniversity of SydneySydneyNew South WalesAustralia

**Keywords:** MRI, MRI‐Linac, MRI‐simulator, radiation therapy, radiography

## Abstract

The increased utilisation of magnetic resonance imaging (MRI) in radiation therapy (RT) has led to the implementation of MRI simulators for RT treatment planning and influenced the development of MRI‐guided treatment systems. There is extensive literature on the advantages of MRI for tumour volume and organ‐at‐risk delineation compared to computed tomography. MRI provides both anatomical and functional information for RT treatment planning (RTP) as well as quantitative information to assess tumour response for adaptive treatment. Despite many advantages of MRI in RT, introducing an MRI simulator into a RT department is a challenge. Collaboration between radiographers and radiation therapists is paramount in making the best use of this technology. The cross‐disciplinary training of radiographers and radiation therapists alike is an area rarely discussed; however, it is becoming an important requirement due to detailed imaging needs for advanced RT treatment techniques and with the emergence of hybrid treatment systems. This article will discuss the initial experiences of a radiation oncology department in implementing a dedicated MRI simulator for RTP, with a focus on the training required for both radiographer and RT staff. It will also address the future of MRI in RT and the implementation of MRI‐guided treatment systems, such as MRI‐Linacs, and the role of both radiation therapists and radiographers in this technology.

## Background

Imaging has always played a significant role in radiation therapy (RT) for volume delineation and localisation as well as treatment planning. Highly conformal treatment techniques require precise definition of tumour and normal tissues to minimise toxicity and maximise the effects of RT on tumour cells. The advancement of treatment technologies has required an improvement in imaging for better soft tissue visualisation of tumour and organs‐at‐risk (OARs).

Magnetic resonance imaging (MRI) has been shown to provide valuable additional information for many tumour sites and associated normal tissues due to its excellent soft tissue discrimination and functional information. The advantages of using MRI for radiotherapy treatment planning (RTP) have been well established for many tumour sites including the brain, head and neck, breast, prostate and cervix.^1^

Despite its advantages, the use of MRI for RTP is limited mainly due to geometric distortion and the absence of electron density information. Cost and lack of availability to patients requiring RT is also a limitation. To mitigate distortion, sequence parameters are modified and distortion correction algorithms are used. The absence of electron density information is overcome by co‐registering MR images to computed tomography (CT) images. However, use of MRI only for radiotherapy planning is being investigated.^2^, ^3^, ^4^ Dedicated MRI simulators located within a radiation oncology department are rare in Australia. A recent Australian survey reported that while 71% of participants had access to diagnostic MRI, only two RT centres had access to a dedicated MRI simulator specifically for RT purposes.^5^ The survey also found that diagnostic MRI and planning MRI were co‐registered with RTP CT by 95% and 34% of participants, respectively.

The adaptation of RT workflow to accommodate MRI has been discussed extensively 5 with a focus on the difference in imaging requirements between RT and diagnostic scanning. Diagnostic MRI refers to scans performed for diagnostic or staging purposes where image quality is more important than patient set up and geometric accuracy. Planning MRI refers to scans performed in the RT treatment position on a flat couch top, with associated immobilisation devices, where maintaining geometrical integrity is of utmost importance. Optimal image quality for volume delineation is also important.

Advances in RT technology have the potential to impact on current workflow and practice and require upskilling in the use of new technologies. Traditionally, the role of radiographers is limited to a diagnostic setting where they are responsible for optimising imaging for diagnosis. In contrast, the role of radiation therapists is in the planning and treatment of patients once a diagnosis has been made.

While the new workflow of MRI utilisation in RT has previously been addressed, there has been little discussion on staff training and integration of such technologies in a standard radiation oncology department.^6^, ^7^, ^8^ This paper will discuss the implementation of a MRI simulator at Liverpool Cancer Therapy Centre with a focus on the radiation therapist and MRI radiographer roles and the consideration of staff needs for MRI‐guided therapy systems.

## Considerations for the Integration of MRI in RT

Liverpool Cancer Therapy Centre installed Australia's first dedicated MRI simulator in June 2013. The MRI Simulator installed is a wide‐bore 3 Tesla Siemens Skyra (Magnetom, Erlangen, Germany), to be utilised for both clinical and research patients. The utilisation of the simulator has been steady since installation, with 26% and 25% of all new case patients in the department requiring a clinical MRI planning simulation for their treatment in 2015 and 2016 respectively.

The implementation of a dedicated MRI simulator^9^ required the involvement of specialised staff, such as a MRI radiographer and MRI physicist, to assist in the set up of the MRI and education of oncology staff. The physicist's role within our team is to provide guidance and knowledge to fellow medical physicists as well as initial training in MRI theory and safety. The MRI radiographer is responsible for the day‐to‐day running of the MRI simulator, as well as liaising with physicists and oncologists for protocol development per tumour site. To ensure that the images acquired using the MRI conform to RTP requirements, radiation therapists play a vital role in communicating the RT specific needs to the radiographers. Each radiation therapist spends time in MRI working with the MRI radiographers to develop an interdisciplinary team with specialised skills in MRI simulation. This created a new specialist RT‐MRI consultant role, recognising the achievements of radiation therapists who undergo postgraduate study in the field of MRI. This fusion of expertise has not only provided invaluable resources and knowledge in the department, but has also allowed for career progression and development for both radiographers and radiation therapists alike.

### RT‐specific requirements

Radiation therapy places additional demands on MRI compared to diagnostic radiology in terms of quality assurance (QA) in order to maintain consistency and accuracy of images and to avoid errors.^10^ A major issue for the integration of MRI into RTP is patient positioning. Patients require reproducible positioning throughout the course of their treatment. Therefore, positioning aids, such as vacuum bags and thermoplastic mask systems, are routinely used in RT imaging. These RT‐specific tools and immobilisation devices present a series of challenges with the introduction of a dedicated MRI simulator. These challenges include modified scanning techniques, different coil arrangements and choice of sequences to maintain the balance between image quality and patient comfort. We installed a wide bore MRI to ensure that the RT‐specific immobilisation equipments, such as coil bridges and larger whole body vacuum bags, are able to fit inside the bore of the MRI. This needed to be considered as some RT immobilisation equipment, such as breast boards with an incline, have physical limitations and may not fit inside the MRI bore.

The inclusion of RT‐specific immobilisation equipment, such as knee and ankle immobilisation, indexing bars, wing board, coil bridges, thermoplastic masks, vacuum bags, etc., is necessary to replicate the treatment position of patients. A flat table top is a RT specific necessity. We use a MRI compatible flat table top by Civco Medical Solutions (MTM3002, Orange City, IA) which is required for RT set up and patient positioning. This improves reproducibility of patient position resulting in increased accuracy of image registration for treatment planning. However, we have shown in a previous study that this has the disadvantage of decreasing signal‐to‐noise ratio (SNR) by over 40% and hence reducing image quality due to the added distance between the coil and patient.^9^ To maintain an acceptable level of image quality for tumour site groups that require imaging with a flat tabletop such as pelvis and head & neck, MRI parameters have been optimised to preserve anatomical detail in imaging required for RTP. Optimisation includes larger field of view (FOV), greater signal averages and changing slice thickness from 2 mm (used in CT simulation) to 3 mm for greater SNR.

The integration of these devices in the department presents two challenges – MRI compatibility of these devices and reduction in image quality due to coils being placed further away from the patient to allow for immobilisation. Batumalai et al.^11^ showed that supine imaging of the breast with a RT‐specific breast board using a 18‐channel surface coil compared to the conventional 16‐channel prone breast coil used in a diagnostic setting, showed a decrease of 41% and 45% in SNR for supine flat and supine inclined (10°) positions respectively.

Basic concepts, such as patient positioning for diagnostic MRI scans, are no longer applicable in the RT department and have to be modified to replicate CT RTP position to allow greater accuracy of image registration in the RT treatment planning system (TPS).

**Figure 1:**
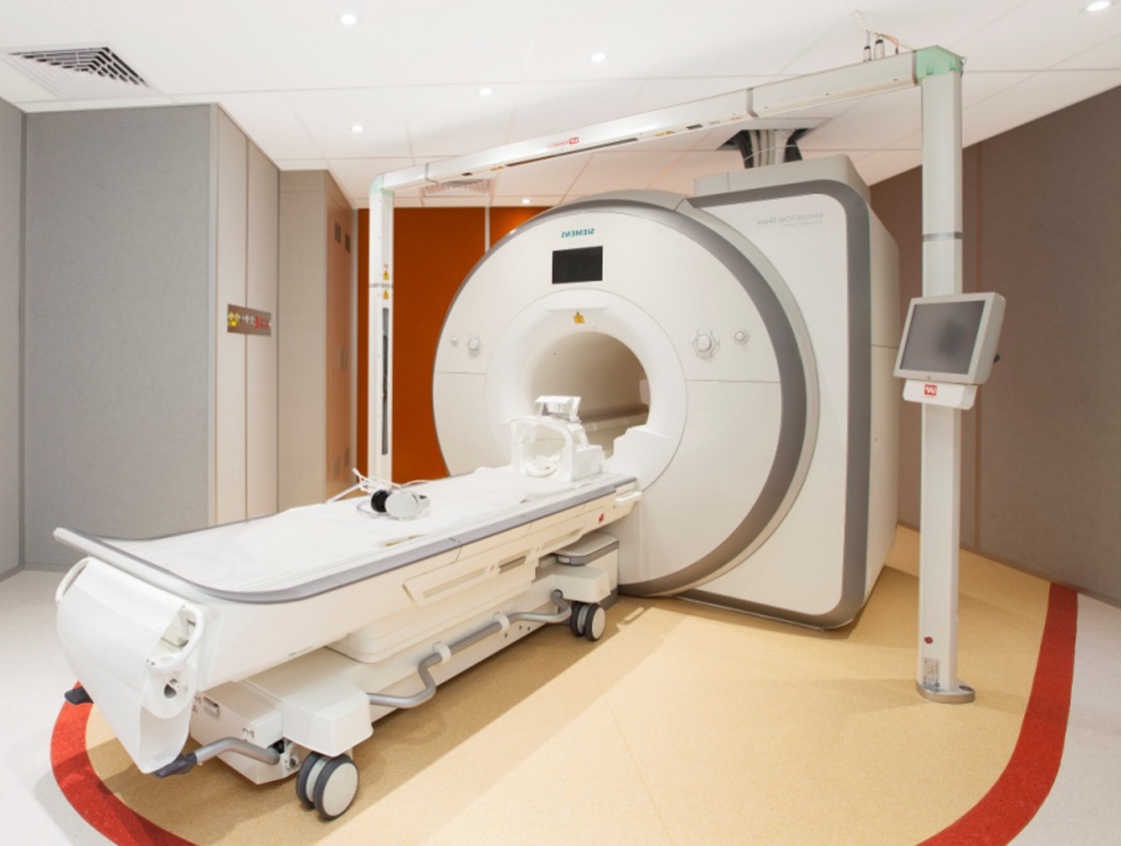
3T Siemens Skyra with external laser positioning laser bridge and marked 30 gauss line (in red) on the floor.

Other considerations include the installation of a RT laser positioning system (Fig. 1) to assist with patient alignment to their treatment tattoos when positioning for MRI scans. The RT laser is used to set up the patient, whereas the MRI bore lasers are used to define the isocentre of the imaging volume. This ensures the same position is replicated in both CT and MRI and further aids in image fusion of the two scans.

Standard MR imaging is not ideal for RTP due to geometric inaccuracies, different patient positioning and magnetic field distortions inherently present in MR images. If these distortions are not corrected and minimised, they have the potential to significantly affect treatment doses to the patient as a result of inaccurate volume delineation.^12^ This presents a challenge in terms of parameter selection when establishing MRI protocols for RTP purposes.

It is also important to ensure the isocentre of slice positions is duplicated for all sequences in RT imaging protocol to streamline the image registration process and minimise errors from misregistration. By maintaining the same slice positions for all scans, the CT‐MRI registration of one MRI dataset can be transferred to all other datasets.

Our centre has developed a QA program for the MRI simulator which was reported by Xing et al.,^9^ based on measurements made with the ‘all‐in‐one’ MagIQ phantom (Leeds Test Objects) during the commissioning period of the scanner. The QA program comprises of daily, monthly, quarterly and annual QA. The daily QA is performed by the radiation therapists and radiographers using a laser alignment phantom (Aquarius Phantom, LAP Laser, Boynton Beach, FL, USA) filled with copper sulphate to test the laser alignment as well as image quality of the scanner daily. Each clinically utilised coil is tested on a monthly basis, RT workflow is tested quarterly and geometric distortion and system performance is tested annually by an MRI physicist using the laser and MagIQ phantoms.

### Protocol development

In the initial phases of MRI implementation, radiologists provided advice on optimal imaging sequences for each tumour site as well as providing advice on imaging quality and target volumes during the planning stage of treatment. However, radiologist support is not always available and creating RT‐specific protocols with minimal radiologist support is a major challenge faced by MRI radiographers in a RT department. Although there are hardware solutions for RT dedicated MRI systems, such as external laser bridges and MRI‐compatible immobilisation devices, there is still a gap in RT‐specific imaging protocols and parameters. This requires the radiographers to closely monitor image quality and protocols with the assistance of medical physicists to ensure that images are acceptable for RTP requirements. Specific parameters must be kept in mind when creating new protocols to minimise inherent geometric distortions. This is important because distortion in MR imaging may result in inaccurate representation of tumour volume thus impacting on dose calculation and quality of treatment delivered to patients.

Other important parameters needed for MRI simulation include large field‐of‐view imaging to allow for image registration to planning CT, high receiver bandwidths to minimise chemical shift and susceptibility‐induced spatial distortion, as well as thin slices and high‐order shimming. These parameter restrictions tend to reduce image quality as a tradeoff, which would not be acceptable in a radiology setting for diagnostic purposes, however is sufficient for RTP purposes.^10^, ^13^ For patients with any metal implants, such as joint prosthesis’ and fiducial markers, metal artefact reduction sequences have been developed to further reduce distortion. Our centre uses WARP (Siemens, Erlangen, Germany), a vendor specific metal artefact reduction sequence comprised of higher receive and transmission bandwidths and view angle tilting (VAT). It assists in minimising paramagnetic susceptibility artefacts while maintaining adequate soft tissue detail close to the metal implants common in oncological imaging such as fiducial markers in the prostate and liver.

With new knowledge of imaging requirements in RT, the radiographers have the added task of training radiation therapists to use the MRI simulator while ensuring they maintain imaging standards for all tumour sites. This has been achieved in our clinical setting through detailed documentation and protocols for each tumour site with an emphasis on RTP requirements.

**Figure 2:**
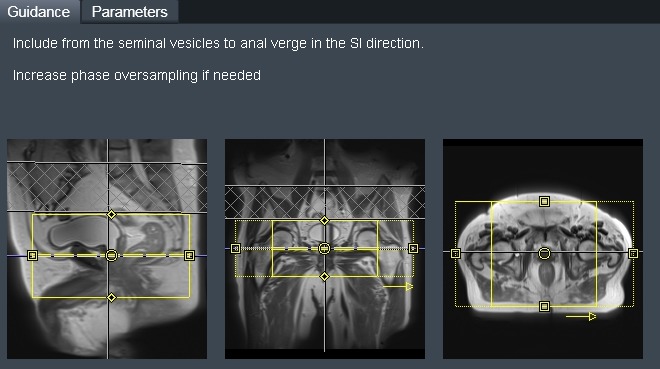
On‐line scanning guidance notes for prostate radiotherapy planning, including anatomical and organs‐at‐risk structures.

**Figure 3:**
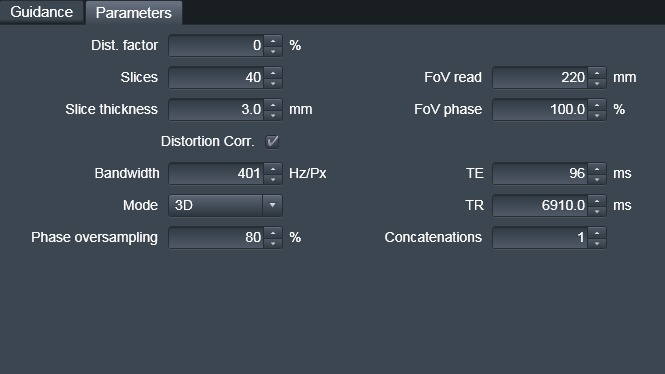
Parameter card includes all relevant radiotherapy treatment planning related parameters that must be verified before scanning.

To address these imaging and training needs, the radiographers have designed an inline semiautomated workflow^14^ on the MRI simulator with integrated scanning instructions for all tumour site groups as set by the radiation oncologists (Fig. 2). This workflow (Figs. 2 and 3) was designed to address the specific needs of the department and the training requirements for radiation therapists with a user‐friendly interface designed for training and standardisation of all imaging protocols. This ensures that all MRI protocols meet RTP requirements for both target volume and OAR coverage (Fig. 2).

MRI parameters are limited to what is required for RTP to minimise geometrical distortions, however, with some flexibility for in parameters to be adjusted for patient‐specific factors such as FOV, number of slices, repetition time and echo time (Fig. 3).

The semiautomated workflow has been successfully implemented at our centre over the previous 2 years and has helped reduce protocol variation and improve imaging for each tumour site. The workflow has decreased scan time for patients and improved radiation therapists understanding of MRI scanning and expedited training in the department. It has delivered improved image reproducibility and increased the confidence of radiation therapists unfamiliar with MRI.

### Education and training

To facilitate the integration of radiographers into a RT department, it was necessary for the radiographers to have a better understanding of the RT work process. This involved rotations in CT simulation, CT‐MRI image registration in the RT TPS and training in volume delineation. Radiographer rotation in CT simulation provided an understanding of basic and advanced treatment set ups to ensure these were accurately replicated in the MRI simulation environment. Using their expertise in diagnostic CT as well as MRI, radiographers were able to contribute to optimisation of the CT simulation protocol including patient preparation and contrast administration. Moreover, training in CT simulation allows for integration of both imaging modalities into one department as opposed to separate entities. This allows for a streamlined workflow by decreasing time lag in imaging between the two modalities, reducing the chance of anatomical variations and allowing for greater consistency between scans. Training in anatomical contouring on the RTP systems gives the radiographers an appreciation of how the MR images are utilised in planning allowing for greater optimisation of imaging parameters to better suit the requirements for RTP.

Radiation therapists were encouraged to attend workshops and conferences to gain a better understanding of the basics of MRI as well as more comprehensive in‐house training organised by MRI radiographers. Radiation therapists rotating through MRI simulation will be expected to demonstrate an understanding of the general MRI processes including departmental quality assurance, safe operation of the MRI equipment, preparation and post‐processing of images for all clinical scans. The intent is to develop the imaging skills of the radiation therapists while allowing them to understand the principles and concepts involved in MRI.

The second component of radiation therapist training focuses on advanced scanning techniques required for research. This has a greater emphasis on the principles of MRI scanning including correct sequence selection for clinical and research requirements, tissue weightings and their application in oncological imaging. A radiation therapist at this level will be able to troubleshoot and overcome common MRI issues including recognition of common artefacts and how to minimise them, advanced scanning including cardiac MRI, diffusion, perfusion and spectroscopy, as well as being able to run daily scheduled activities without the assistance of a radiographer.

### Safety

#### Staff

MRI safety is another challenge with the biggest concern being staff who are unaccustomed to working in a high magnetic field environment. Education of staff including medical physicists, radiation therapists, nurses and oncologists is required to ensure protection of both patients and staff working in the MRI department. Safety presentations are run yearly in our department to stress the importance of safe practice in MRI. This covers the dangers the MRI can pose, such as projectile capabilities of common ferromagnetic objects, as well as screening of patients for contraindications such as cardiac pacemakers and neurostimulators. Although MRI safety is understood well in a radiology setting, we found that the dangers of MRI are not commonly understood in an oncology department. We therefore have a structured MRI safety program to ensure staff access to the area is only granted upon satisfactory completion of MRI safety competencies. Moreover, the MRI simulator room has a red demarcation line integrated in the floor design (Fig. 1) indicating the 30 gauss line so staff are aware of the dangers of ferromagnetic objects once that line has been crossed.

#### Patient

A comprehensive pre‐MRI screening of patients is performed by the Radiation Oncologists prior to their scan booking. Our centre follows The Royal Australian and New Zealand College of Radiologists (RANZCR) guidelines for administration of gadolinium.^15^ The centre's policy is to not scan patients with pacemakers irrespective of their conditional compatibility. If there is any doubt about the MR compatibility of other implantable devices in a patient, we err on the side of caution and do not scan the patient, relying on the CT alone for volume delineation. A full appraisal of patient and implant safety can be found in the following references.^16^, ^17^

## Future Directions

While imaging for RTP has evolved to include enhanced soft tissue detail and functional information, so have the imaging requirements for treatment verification. We have seen a shift from standard planar imaging to multiparameteric imaging such as MRI for added soft tissue contrast, physiological information and more specifically MRI‐guided treatment. MRI‐guided systems allow verification of treatment in real time and monitoring of tumour response, both anatomically and functionally.

**Figure 4:**
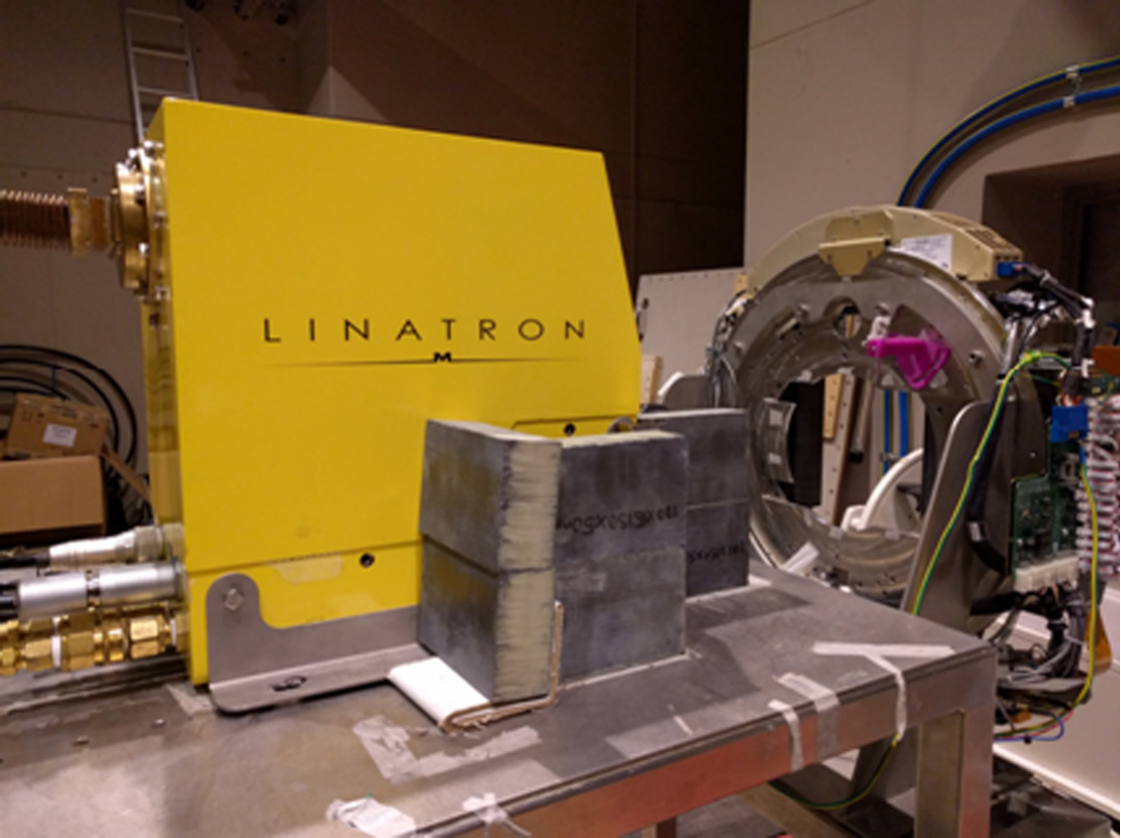
Fixed gantry linatron with multileaf collimators (MLCs) (white arrow) outside the magnetic resonance imaging faraday cage. The bore is positioned that the main magnetic field is aligned parallel with linatron beam.

The development of a linear accelerator with an integrated MRI provides the potential for advanced image guidance techniques. Collaboration and cross‐training between RT and medical imaging is thus vital. This will allow optimal utilisation of any MRI‐d system in terms of making appropriate imaging decisions and troubleshooting in a timely fashion for adaptive treatments. The Ingham Institute for Applied Medical Research along with other research groups is in the process of developing a 1 Tesla MRI‐guided linear accelerator (Appendix 1). Liverpool Cancer Therapy Centre currently houses Australia's first prototype MRI‐Linac driven by the Ingham Institute. The system is in its second phase of testing with recent installation of a unique split‐bore magnet and Linatron (Fig. 4). Although the Australian system is still in the testing phase, ViewRay have an MRI‐guided cobalt system available for patient treatments^18^ and it is expected that the first patient treatments with an Elekta MRI‐Linac system will occur in the near future.^19^ The radiographers and radiation therapists at Liverpool Cancer Therapy Centre have been involved in the MRI‐Linac project including the initial design phases of the patient rotation device^20^ and imaging on the first prototype design.^21^ The role of radiation therapists and radiographers in this technology is paramount to the streamlined integration of these systems in clinical practice.

## Discussion

The role of radiographers, as well as radiation therapists has evolved significantly with the implementation of MRI simulation and the increased use of MRI for treatment planning. Radiographers in our department have been trained to understand the basic concepts of RT as well as educate radiation therapists on the basics of MRI and safety as a first step for integration. Having no previous experience working in RT, MRI radiographers had to use their knowledge in medical imaging to adjust patient set up, scanning parameters and build RT‐specific protocols so that images can be used for fusion and treatment planning purposes.

Radiation therapists are accustomed to evaluating CT and positron emission tomography images for planning and cone‐beam CT and electronic portal images for verification of patient position during treatment. The introduction of MRI in the department has allowed the radiation therapists to become familiar with a new imaging modality for RTP, particularly in understanding the differences in MRI techniques and sequences. This is particularly advantageous with the greater utilisation of MRI for planning and for image guidance during RT treatment. We believe the transition of radiation therapists to use a MRI‐Linac system will be made easier with their prior knowledge of MRI simulation including image quality, protocols, safety and quality assurance. Day‐to‐day scanning and operation of the MRI‐Linac will require a minimum of two staff members who have undergone the MRI safety training package and possess an EPA licence to be able to scan and treat patients.

However, it should be noted that the QA and safety program, as well as image quality will not be directly translatable from the MRI Simulator to the MRI linear accelerator (Linac). When the MRI‐Linac is operational to treat patients a multidisciplinary group will need to create safety guidelines taking into account safety issues for both the MRI and Linac. The safety guidelines will take into account the 5 gauss line which extends beyond the Faraday cage unlike standard MRI configurations. QA will also differ to the MRI simulator as it will need to incorporate QA of the Linatron as well as the MRI and notably the configuration and alignment of the two devices together.

Due to the differences in the magnetic field strength and gradient linearities of the MRI Simulator and the MRI‐Linac, we anticipate that the MRI‐Linac will have poorer image quality and have greater spatial distortion, which will need to be accounted for when building imaging protocols. Although this is a limitation of the MRI‐Linac system compared to the MRI Simulator, it is a challenge that will be addressed in the future with further investigation.

The integration of MRI simulation in our department has allowed radiation therapists to build a greater understanding of the basics and concepts of MRI which may not always be covered during formal education training. This stresses the need for formal qualifications and accreditation for radiation therapists in the field of medical imaging as a pivotal part of the profession.

The increasing use of MR imaging in RTP and treatment has highlighted the need for greater multidisciplinary collaboration in the field of medical radiation sciences as hybrid technologies provide many advantages in the field of treatment. As the demand for more complex image guidance in RT increases, the future direction of the profession needs to evolve to keep up with advances in technology.

## Conclusion

Implementation of a dedicated MRI scanner in our department has presented a number of challenges with a balance achieved between optimal image quality and minimal geometric distortion. This process has required close collaboration between MRI radiographers and radiation therapists with their differing skill sets. For the full potential of MRI in RT to be harnessed, additional cross‐training and skill expansion between MRI radiographers and radiation therapists will be necessary, providing a gateway for improved clinical outcomes and further research.

## Conflict of interest

The authors have no conflict of interest to declare.

## Appendix 1 Summary table of current MRI‐Linac systems

**Table:**

MRIgRT system	Magnetic field strength (T)	Bore Size (cm)	Linac energy	B0 field direction	Rotating couch/rotating gantry	Advantages/disadvantages
MRI on rails^1^	1.5	70 closed bore	6 MV	NA	NA	Advantage – High‐quality MR imaging, high‐quality treatment delivery, including non‐coplanar beam angles Disadvantage – No real‐time imaging
ViewRay^2^	0.35	70 closed bore	3× ^60^Co sources	Perpendicular	Rotating gantry	Advantage – Multiple systems treating patients. ^60^Co decay interferes less with MRI unit. Low‐field MRI has minimal impact on dose distribution. Low strength MRI has minimal susceptibility artefact, hence limited image distortion. Disadvantage – Low‐field MRI limited to anatomical imaging, ^60^Co has limited beam penetration and slightly larger field penumbra.
Rotating Biplanar MRI‐ Linac^3^	0.5	85 open bore	85 open bore	Parallel or perpendicular	Rotating gantry	Advantage – Biplanar system allows beam configuration at both parallel and perpendicular orientation. Low magnet strength minimises radiation hotspot at the interface of lung and tissue. Disadvantage – Image quality at low field
UMC Utrecht^4^	1.5	70 closed bore	6 MV ring mounted gantry	Perpendicular	Rotating gantry	Advantage – Broad group of clinical collaborators and advanced development. Close to clinical MRI system with high image quality. Disadvantage – To manage the electron return effect. Irradiation through the cryostat.
Australian MRI‐LINAC system^5^	1	82 open bore	6 MV fixed gantry	Parallel and perpendicular	Rotating couch	Advantage – High‐field inline system with flexibility to investigate different beam‐magnet orientations. Dosimetric advantages for small lung targets. Static gantry allows for more compact design of the system. Disadvantage – Impact of patient rotation requires further investigation in regards to tissue deformation and patient comfort.

MRIgRT, MRI‐guided radiation therapy; T, tesla; B0, main magnetic field; MRI, magnetic resonance imaging; Co, cobalt; Linac, linear accelerator; MV, megavoltage; UMC, University Medical Centre.
